# A preliminary animal study of thermal rheology fluid as a new temperature-dependent liquid intravascular embolic material

**DOI:** 10.1007/s11604-021-01232-3

**Published:** 2021-12-01

**Authors:** Yugo Imai, Shobu Watanabe, Norihisa Nitta, Shinichi Ota, Shigeru Yao, Yoshiyuki Watanabe

**Affiliations:** 1 Department of Radiology, Shiga University of Medical Science, Seta Tsukinowa-Cho, Otsu, Shiga 520-2192 Japan; 2 Department of Radiology, National Hospital Organization, Higashi-Ohmi General Medical Center, Higashiomi, Shiga Japan; 3 Department of Radiology, Kyoto Okamoto Memorial Hospital, Kyoto, Japan; 4 Department of Radiology, Nagahama Red Cross Hospital, Nagahama, Shiga Japan; 5 Research Institute for the Creation of Functional and Structural Materials, Fukuoka University, Fukuoka, Japan

**Keywords:** Thermal rheology fluid, Intravascular embolic material, Transarterial embolization, Animal study

## Abstract

**Purpose:**

Thermal rheology (TR) fluid, which comprises polyethylene (PE) particles, their dispersant, and solvent, is a material that increases in viscosity to various degrees depending on the type and ratio of these constituents when its temperature rises. The viscosity of type 1 (TRF-1) increases more than that of type 2 (TRF-2) near rabbit body temperature. This preliminary animal study aimed to determine the basic characteristics and embolic effect of TR fluid by comparing TRF-1 and TRF-2.

**Materials and methods:**

Twenty-four Japanese white rabbits underwent unilateral renal artery embolization using TRF-1 or TRF-2 and follow-up angiography at 7 or 28 days (4 subgroups, *n* = 6 each). Subsequently, the rabbits were euthanized, and the embolized kidneys were removed for pathological examination. The primary and final embolization rates were defined as the ratio of renal artery area not visible immediately after embolization and follow-up angiography, respectively, to visualized renal artery area before embolization. The final embolization rate and maximum vessel diameter filled with PE particles were compared between materials. Moreover, the embolic effect was determined to be persistent when a two-sided 95% confidence interval (CI) for the difference in means between the embolization rates was < 5%.

**Results:**

The final embolization rate was significantly higher for the TRF-1 than for the TRF-2 at both 7 (mean 80.7% [SD 18.7] vs. 28.4% [19.9], *p* = 0.001) and 28 days (94.0% [3.5] vs. 37.8% [15.5], *p* < 0.001). The maximum occluded vessel diameter was significantly larger for TRF-1 than for TRF-2 (870 µm [417] vs. 270 µm [163], *p* < 0.001). The embolic effect of TRF-1 was persistent until 28 days (difference between rates − 3.3 [95% CI − 10.0–3.4]).

**Conclusion:**

The embolic effect of TRF-1 was more persistent than that of TRF-2, and the persistency depended on the type and ratio of TR fluid constituents.

## Introduction

**Fig. 1:**
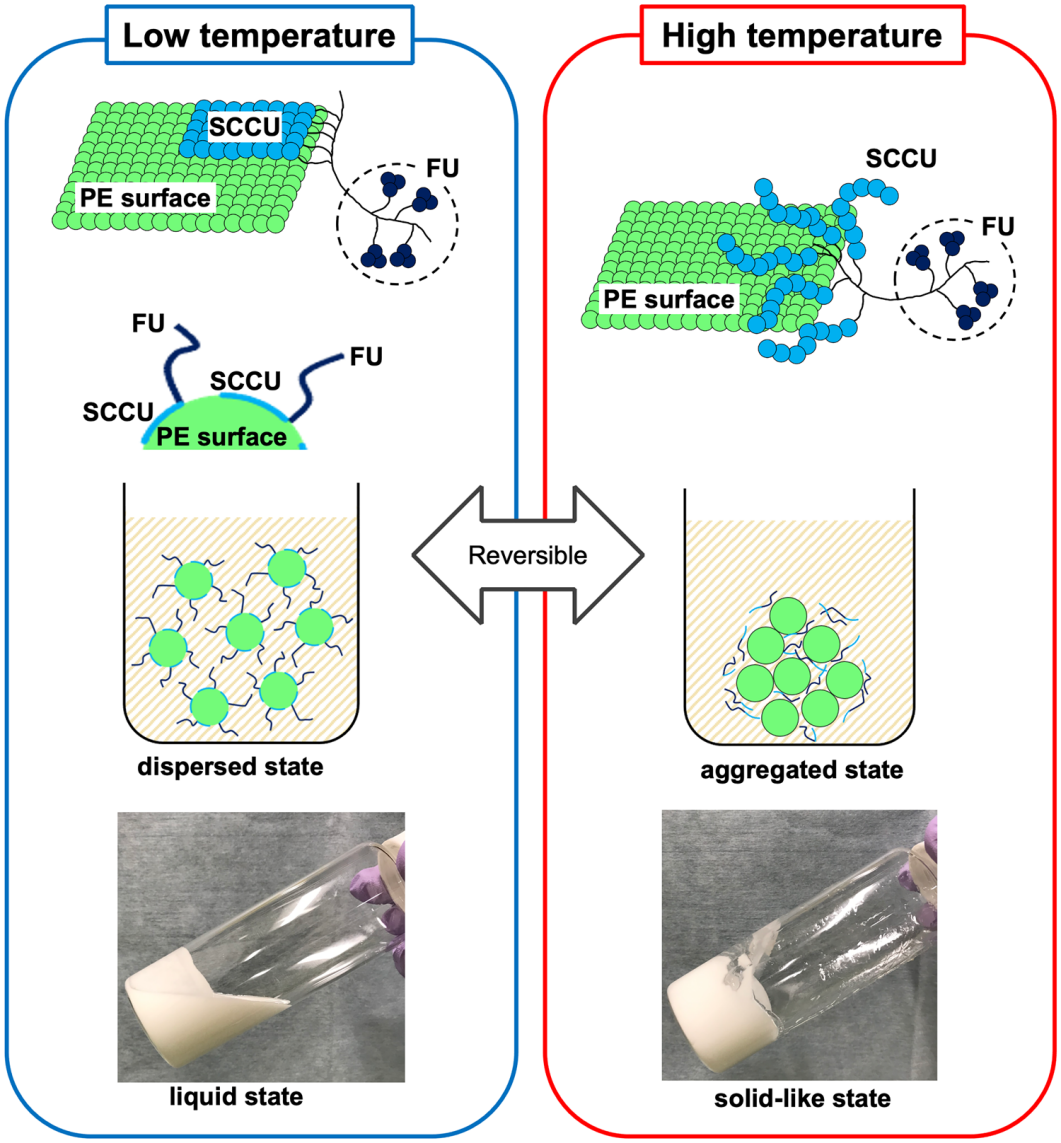
Schematic of the mechanism of the thermal rheology effect and the appearance of thermal rheology fluid. At a temperature lower than the transition temperature, the side-chain crystalline block copolymer (SCCBC) is attracted to polyethylene (PE) surface by van der Waals forces and is epitaxially arrayed to the PE surface because of structural similarity of the side-chain crystalline unit (SCCU) of SCCBC to PE. The strong adsorption of SCCBC on the PE surface is caused by cocrystal formation. As a result, the functional units (FUs) of SCCBC cover the PE surface and form a new layer, which precludes aggregation between PEs; hence, the thermal rheology (TR) fluid exists in this state as a low-viscosity fluid. On the other hand, at a temperature higher than the transition temperature, the crystalline part of SCCBC melts, van der Waals forces disappear, and SCCBC is desorbed from PE surface. As a result, PE regains its original properties and aggregates; the TR fluid increases in viscosity and turns into almost solid state. Viscosity changes dramatically and reversibly near the melting point of SCCBC

The side-chain crystalline block copolymer (SCCBC), a dispersant that reduces the viscosity of a concentrated polyethylene (PE) particle suspension, was developed by Yao et al. [1–6]. The SCCBC consists of a long alkane side-chain unit that can be adsorbed on the crystalline surface of PE particles via van der Waals forces and a functional unit (FU) with solvent affinity. Therefore, by the action of the SCCBC, a PE particle suspension exists as a low-viscosity fluid; however, as the temperature of the suspension rises above the so-called “transition temperature,” the crystalline part of SCCBC melts, van der Waals forces disappear, and the suspension dramatically increases in viscosity and turns into almost solid state [3, 6]. This temperature-dependent change in viscosity is reversible [1–3, 6]. This phenomenon was named thermal rheology (TR); accordingly, the PE particle suspension containing SCCBC is called TR fluid (Fig. 1) [2, 6]. The transition temperature and the degree of increase in viscosity can be adjusted by changing the type and ratio of TR fluid constituents: the side-chain crystalline unit (SCCU) of SCCBC, the FU of SCCBC, PE particles, and their solvent [2, 5, 6]. TR fluid is prepared without using a special experimental apparatus and consists of low-cost materials [7]; in addition, TR fluid is stable to external stimuli, and its effect is maintained over a long period [6]. Hence, we can repeatedly change the type and ratio of TR fluid constituents to obtain a mixture with the desired function, and the TR fluid can be stored until use. As a result of such trial-and-error, TR fluids that may be suitable as a new embolic material for transcatheter arterial embolization were developed with the following characteristics: transition temperature adjusted to body temperature, and a radiopaque material containing a contrast medium as the solvent. This TR fluid is liquid outside the body but could behave like a solid when injected into blood vessels.

Various embolic materials, including gelatin sponges (GS), trisacryl gelatin microspheres, n-butyl-2-cyanoacrylate (NBCA), and metallic coils, are in widespread clinical use [7]. Although each is associated with distinct strengths, each also has limitations. Thus, the availability of a new embolic material increases the likelihood of better embolic therapy, and it is meaningful to investigate whether a TR fluid with a unique TR effect could be used as an embolic material. Moreover, although Hirai et al. reported that TR fluid could be used as an embolic agent, its detailed characteristics need further elucidation [8].

In this study, we used a rabbit model to determine the basic characteristics and embolic effect of TR fluid by comparing two types of TR fluids with different viscosity-temperature relationships: types 1 (TRF-1) and 2 (TRF-2).

## Materials and methods

### Animals

All experiments were approved by the Animal Experimentation Committee of our institute and conducted in accordance with the Animal Care Guidelines.

A total of 24 Japanese white rabbits (body weight: 2.5–3.0 kg), which were obtained from an animal breeding company (KITAYAMA LABES CO., LTD, Nagano, Japan), were used in this study. The mean rectal temperature of the rabbits was 39.1 °C (SD 0.4). They were housed in our animal room under constant environmental conditions (temperature: 22 ± 1 °C, humidity: 60 ± 5%, 12 h light/dark cycle), and received 150 g rabbit feed (CR-3 M, Clea Japan Inc., Tokyo) daily with clean drinking water ad libitum.

#### SCCBC

**Fig. 2:**
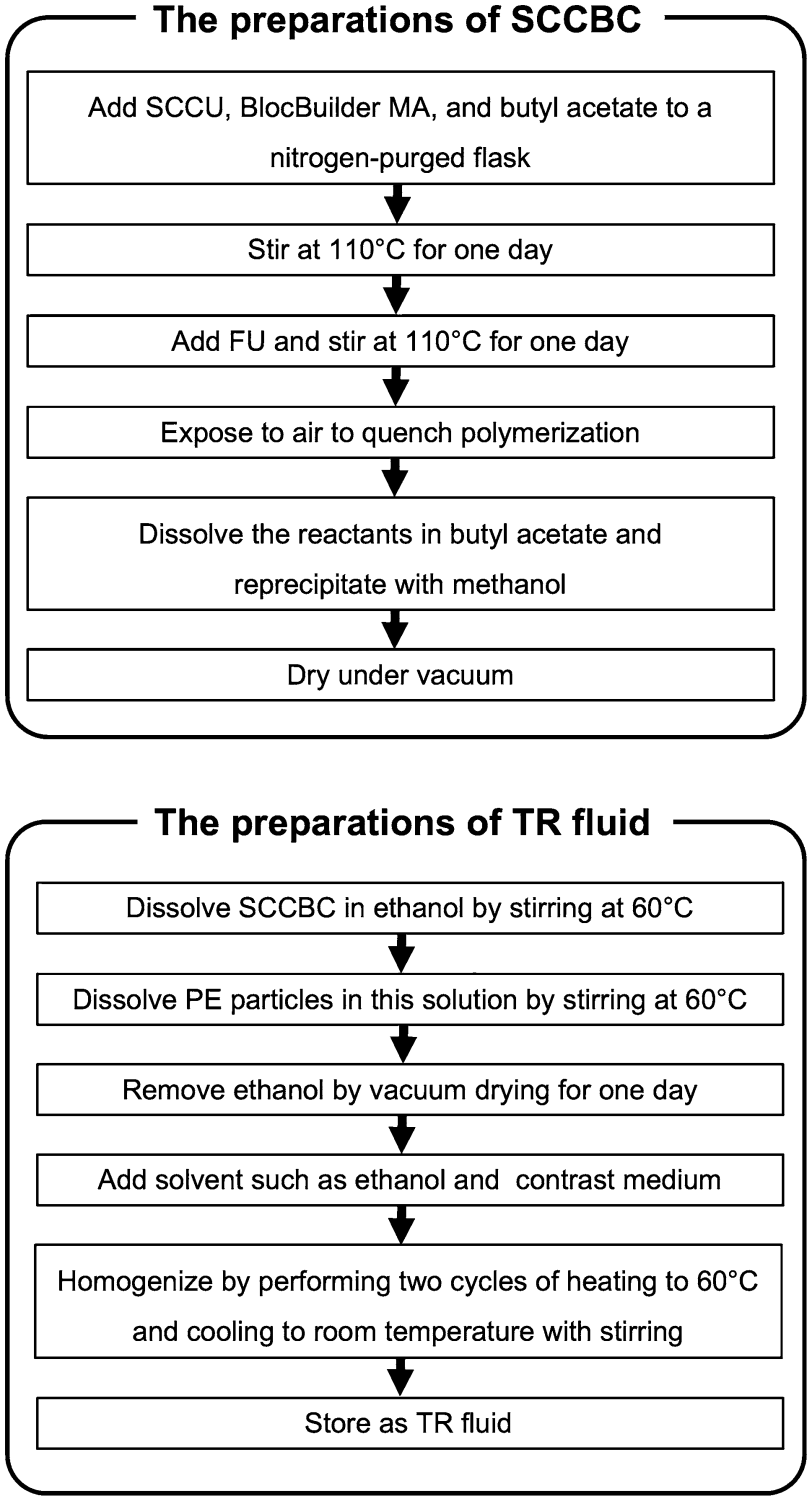
Preparations of a side-chain crystalline block copolymer and thermal rheology fluid. Side-chain crystalline block copolymer (SCCBC) is prepared as follows: first, the side-chain crystalline unit (SCCU), BlocBuilder MA as an initiator, and butyl acetate as a solvent are added to a nitrogen-purged flask. The mixture is then stirred at 110 °C for one day, and a solution of the functional unit (FU) in butyl acetate is added to the mixture and polymerized for one more day. Next, the solution is exposed to air and rapidly cooled to quench polymerization. Finally, the reactants are dissolved in butyl acetate, reprecipitated with methanol and dried under vacuum. Thermal rheology (TR) fluid is prepared as follows: first, SCCBC is dissolved in ethanol by stirring at 60 °C, and PE particles are added to this solution and stirred. Subsequently, the ethanol was removed by vacuum drying for one day. Next, a solvent is added, mixed, homogenized by performing two cycles of heating to 60 °C and cooling to room temperature, and then stored as a TR fluid

SCCBC and TR fluid were prepared and stored under clean conditions. The procedures for preparing these materials are shown in Fig. 2.

Two types of SCCBCs were prepared in this study: SCCBC-1 and SCCBC-2. For SCCBC-1 and SCCBC-2, stearyl acrylate (Tokyo Chemical Industry, Tokyo, Japan) and hexadecyl acrylate (Tokyo Chemical Industry) were selected as SCCUs, respectively. Diethylene glycol ethyl ether acrylate (Sigma-Aldrich Japan, Inc., Tokyo, Japan) and acrylic acid (Tokyo Chemical Industry) were selected as the FUs of SCCBC-1 and -2, respectively. Each SCCBC was synthesized by nitroxide-mediated living radical polymerization, as previously reported [5]. Briefly, the SCCU, 2-methyl-2-[*N*-tert-butyl-*N*-(1-diethoxyphosphoryl-2,2-dimethyl-propyl)aminoxy]propionic acid (BlocBuilder MA, Arkema Inc., Colombes, France) as an initiator, and butyl acetate (Wako Pure Chemical Industries, Osaka, Japan) as a solvent were added to a nitrogen-purged flask. The mixture was then stirred at 110 °C. After 1 day, a solution of the FU in butyl acetate was added to the mixture and polymerized. One day later, the solution was exposed to air and rapidly cooled to quench polymerization. Finally, the reactants were dissolved in butyl acetate, reprecipitated with methanol (Wako Pure Chemical Industries) for purification, and dried under vacuum. Consequently, the white solids SCCBC-1 and -2 were obtained.

### TR fluid

Two different types of TR fluids, TRF-1 and TRF-2, were prepared in this study as these would allow us to assess the viscosity-temperature relationship with changes in the type and ratio of TR fluid constituents. Basically, TRF-1 was made by adding contrast media to the original TR fluid, developed as a dispersant for PE particle suspension. In addition, the constituent ratio was adjusted to increase the viscosity at near rabbit body temperature. TRF-2 was made from the original TR fluid and contained a large amount of contrast media. It did not contain ethanol as solvent; therefore, the FU was changed to maintain solvent affinity. The SCCU type and the TRF-2 constituent ratio were adjusted to increase the viscosity at near rabbit body temperature.

The preparations of TR fluids were as follows: first, SCCBC-1 or -2 was dissolved in ethanol (Wako Pure Chemical Industries) by stirring at 60 °C, and PE particles (average diameter: 7.4 µm, Ceridust 3620, Clariant Japan, Tokyo, Japan) were added to this solution and stirred. Subsequently, the ethanol was removed by vacuum drying for 1 day. The obtained substances were composed of 2% SCCBC and 98% PE particles. Next, the solvent was added, mixed, homogenized by performing two cycles of heating to 60 °C and cooling to room temperature, and then stored as a TR fluid. TRF-1 contained 25% ethanol, 40% contrast medium (350 mg I/mL, Iomeron, Eisai, Tokyo, Japan), and 35% SCCBC-1-bound PE particles; TRF-2 contained 75% contrast medium and 25% SCCBC-2 bound PE particles. SCCBC-1 comprised 0.7% of the total volume of TRF-1, and SCCBC-2 comprised 0.5% of the total volume of TRF-2.

**Fig. 3:**
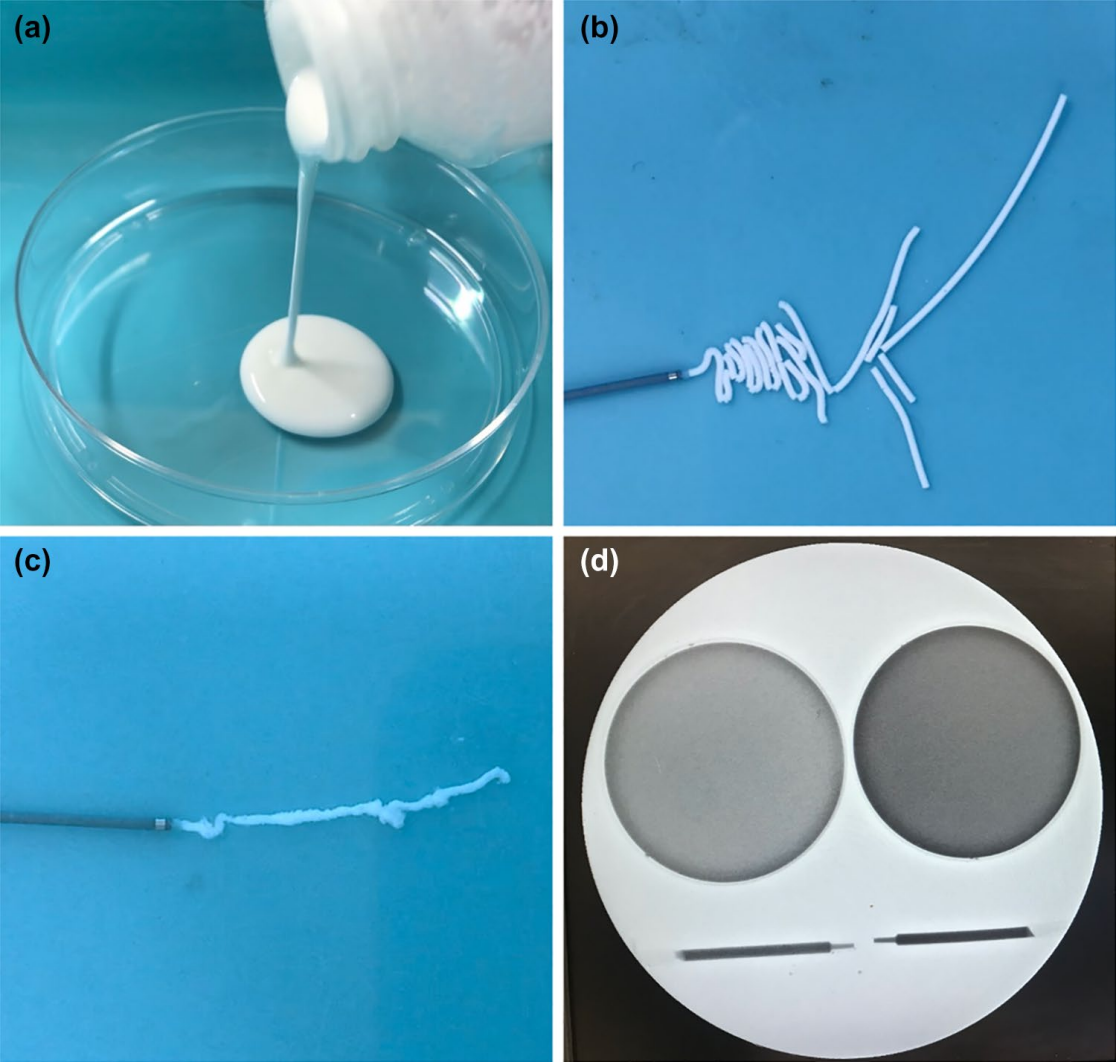
Properties of the thermal rheology fluid. The thermal rheology (TR) fluid (type 1, TRF-1) behaves as a low-viscosity fluid at room temperature of 25 °C (**a**) and is extruded from the microcatheter as a rod-shaped solid body at 40 °C, which is close to rabbit body temperature (**b**). Type 2 TR fluid (TRF-2) appears to be less cohesive than TRF-1 at 40 °C (**c**). Both TRF-1 and TRF-2 (left side and right side, respectively) in the Petri dish (above) and 1-mL syringe (below) show visibility in the fluoroscopic image: the latter is better (**d**)

**Fig. 4:**
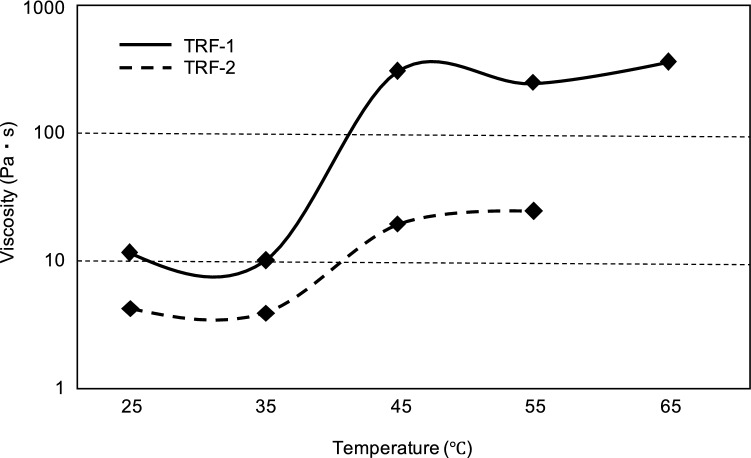
Relationship between the temperature and viscosity of thermal rheology fluid. The viscosity of the thermal rheology (TR) fluid changes dramatically when approaching rabbit body temperature. Note that type 1 TR fluid (TRF-1) has a greater increase in viscosity near body temperature than does type 2 TR fluid (TRF-2)

TRF-1 and TRF-2 were designed so that their viscosity would increase at near rabbit body temperature and be visible under fluoroscopy (Fig. 3). The temperature dependence of the TR fluid viscosity is shown in Fig. 4. The viscosities of the TR fluid at 25, 35, 45, 55, and 65 °C were measured using a cone-plate rheometer (Rheosol-G2000W, UBM Co., Ltd., Kyoto, Japan).

### Embolization

The laboratory room temperature was maintained at 25 °C using an air conditioner. Each rabbit was anesthetized with an intramuscular injection of medetomidine hydrochloride (0.1 mg/kg body weight; Domitor, Zonoac, Fukushima, Japan) and ketamine hydrochloride (20 mg/kg body weight; Ketalar, Daiichi Sankyo, Tokyo, Japan). Once anesthetized, the rabbits were placed supine, and their upper and lower limbs were tied to the operating table.

The right femoral artery was exposed, and a 4-F introducer sheath (Super sheath, length: 7 cm, Medikit, Tokyo, Japan) was inserted into the aorta via a cut-down approach. A 4-F cobra-shaped catheter (Terumo Clinical Supply, Gifu, Japan) was placed into a single renal artery through the introducer sheath, and digital subtraction angiography (DSA) was performed by manual injection of 4 mL of 50% diluted iodine contrast medium (370 mg I/mL, Iopamidol 370, Bayer Yakuhin, Osaka, Japan) using an X-ray fluoroscopy system (Plessart 50 DREX-WIN20P, Canon Medical Systems, Tochigi, Japan). We selected either the right or left renal artery. The radiation parameters used for DSA were as follows: 70 kV output voltage, 50 mA current, and 40 ms pulse width. A 2.2-Fr distal, 2.9-Fr proximal microcatheter (Sniper 2, 110 cm; Terumo Clinical Supply) was then advanced to the tip of the 4-F catheter, and liquid TR fluid collected in a 2.5 mL syringe (Terumo Syringe, Terumo, Tokyo, Japan) was continuously delivered through the microcatheter until either embolization of the renal artery or filling of the segmental arteries and additional peripheral arteries was achieved. TRF-1 and TRF-2 were used for 12 rabbits each. After embolization, DSA was performed again in the same manner, and the catheter and sheath were removed. Subsequently, the femoral artery was ligated.

### Follow-up angiography

The embolized renal artery was catheterized via the left femoral artery, and follow-up DSA was performed. Each group of 12 rabbits were randomly divided into subgroups a and b (*n* = 6 each). Subgroups 1–a and 2–a underwent follow-up DSA 7 days after embolization and subgroups 1–b and 2–b 28 days after embolization. The follow-up period was decided based on reports describing GS particles as a temporary embolic material. It was reported that GS particles completely disappear from the embolized vessels in pathological images within 2–3 weeks, resulting in recanalization [9–11].

### Histopathology

After follow-up angiography, the rabbits were euthanized using pentobarbital (Nembutal, Dainippon Sumitomo Pharma Co., Ltd., Tokyo, Japan), and their embolized kidneys were removed. The specimens were fixed in 10% formalin, embedded in paraffin, cut into 3.5 µm sections, and stained with hematoxylin–eosin for microscopic examination. Pathological images were captured using an Olympus IX83 microscope (Olympus).

### Assessment

**Fig. 5:**
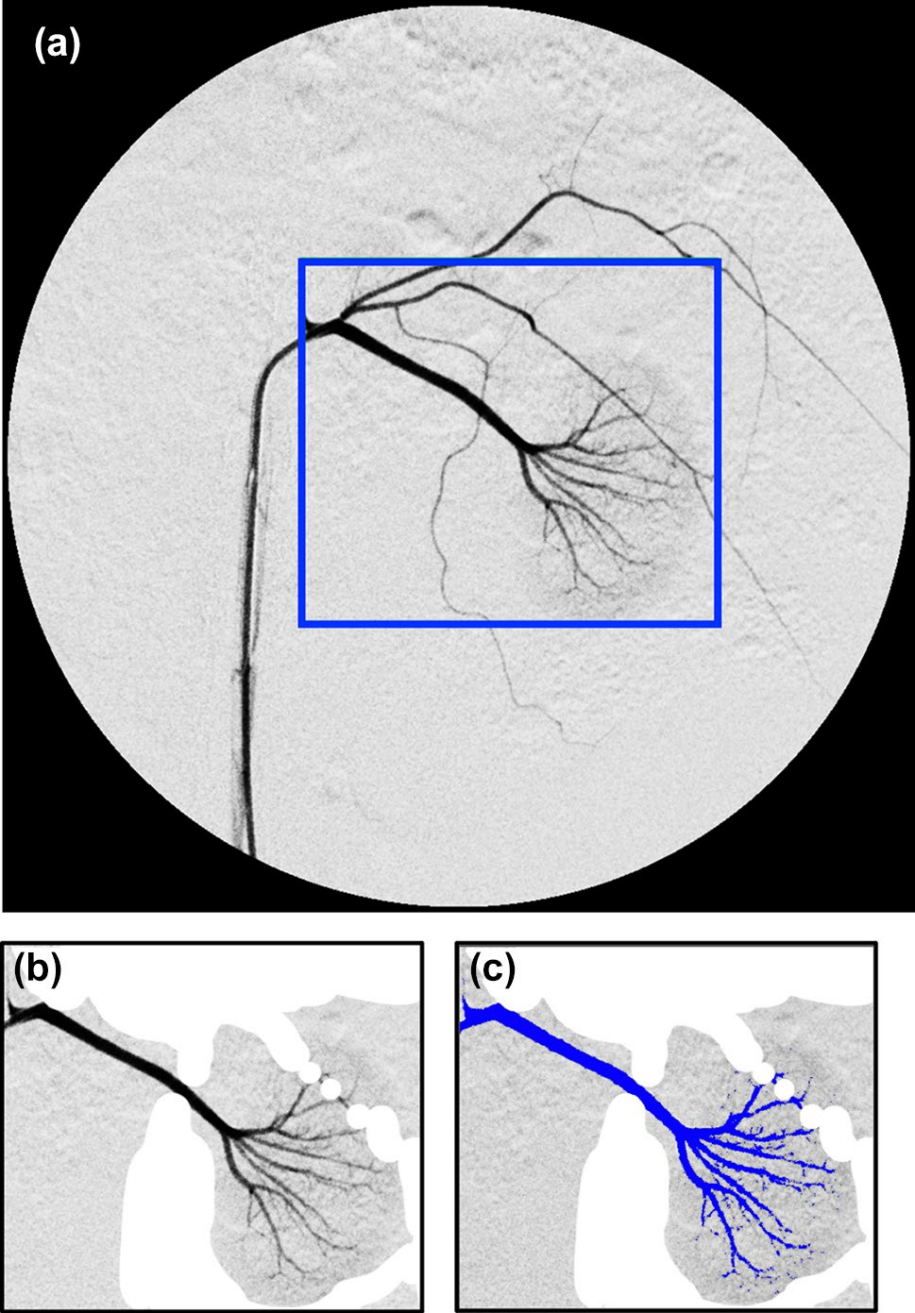
The measurement method of the areas of renal arterial vessels. We selected for measurement an image in which stained tree-shaped renal arterial vessels but not the renal parenchyma was visible (**a**). First, the area of interest (the area bordered by a blue rectangle) was selected (**a**). The extra black areas, such as the renal capsule artery, were masked (**b**). A threshold was then set to distinguish the area of renal arterial vessels filled with contrast media (colored blue) from the background (**c**), and the area was calculated automatically

Angiographic and pathological images were analyzed using ImageJ software, version 1.53a (National Institutes of Health, Bethesda, MD, USA). The areas of the renal arterial vessels visualized during the arterial phase of pre-embolization DSA, post-embolization DSA, and follow-up DSA were measured. Specifically, for each angiography, we selected for measurement an image in which branch-shaped renal arterial vessels, but not renal parenchyma, were stained. The area of interest was selected in each image, followed by masking the extra black areas such as the renal capsule artery (Fig. 5). Next, a threshold was set to distinguish the area of renal arterial vessels filled with contrast media from the background, and the area was calculated automatically. The primary and final embolization rates indicated the embolization rate immediately after embolization and that at the time of follow-up DSA (days 7 or 28), respectively, and were defined and calculated as follows:

$$ {\text{Primary embolization rate }} (\% ) \, = \, \left( {1 - \frac{{{\text{the area of renal arterial vessels visualized on post - embolization DSA}} }}{{\text{the area of renal arterial vessels visualized on pre - embolization DSA}}}} \right) \, \times 100; $$

$$ {\text{Final embolization rate}} \, (\% ) \, = \, \left( {1 - \frac{{\text{the area of renal arterial vessels visualized on follow - up DSA}}}{{\text{the area of renal arterial vessels visualized on pre - embolization DSA}}} } \right) \, \times \, 100. $$

The primary and final embolization rates were compared between subgroups 1–a and 2–a (DSA on day 7) and between subgroups 1–b and 2–b (DSA on day 28). Furthermore, to determine whether the embolic effect was persistent in each subgroup, a two-sided 95% confidence intervals (CI) for the mean differences between the primary embolization rate and final embolization rate, representing the time course of the embolization rate (%), were calculated; when the 95% CI was < 5%, we determined that the embolic effect was persistent, and when the 95% CI was > 15%, the embolic effect was determined to be not persistent.

Pathology was assessed by observing the size and color of the embolized kidneys, distribution of PE particles in the renal arterial vessels, and renal parenchymal changes such as inflammation and necrosis. The maximum vessel diameter filled with PE particles was measured microscopically for each rabbit, and the range of embolization was compared between the TRF-1 and TRF-2 groups.

### Statistical analysis

Continuous data were presented as means with standard deviations (SDs). The Welch *t* test was used for all comparisons. Statistical significance was set at *p* < 0.05. Statistical analyses were performed using IBM SPSS Statistics for Macintosh, Version 25 (IBM Corp., Armonk, NY, USA).

## Results

### Angiographic analysis

**Fig. 6:**
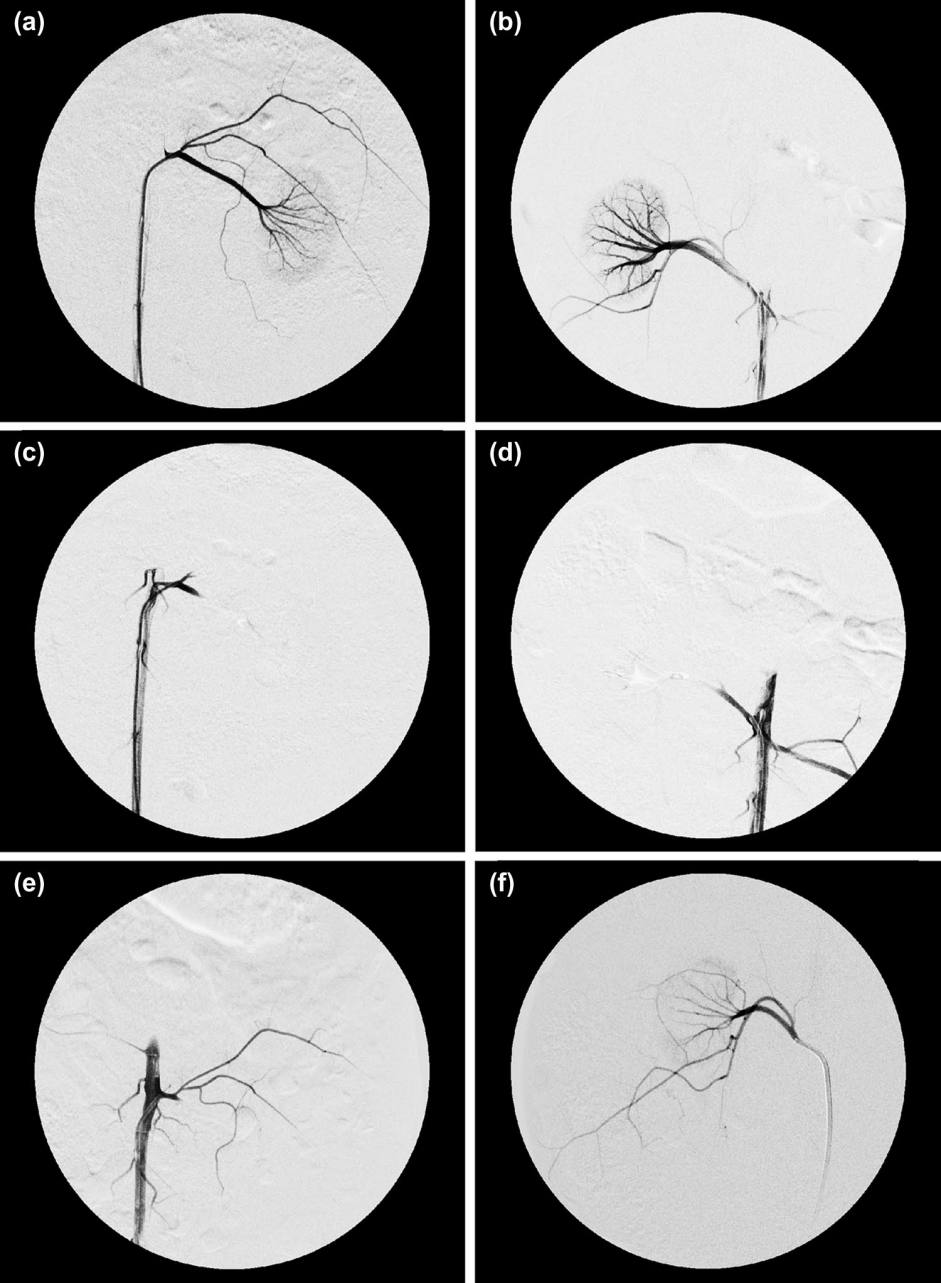
Angiographic findings. In both type 1 and 2 thermal rheology fluids (TRF-1 and TRF-2, respectively) groups, pre-embolization angiography allowed visualization of all branches of the renal artery and renal parenchyma (**a** and **b**, respectively), whereas these were not visible in post-embolization angiography (**c** and **d**, respectively). In TRF-1 group, although the renal capsule artery was visualized, the delineation of renal arterial vessels on angiography at 28 days was similar to that on post-embolization angiography, and no recanalization of the renal artery occurred (**e**). In TRF-2 group, recanalization of the embolized vessels was observed on angiography at 28 days (**f**)

The TR fluid was visible on fluoroscopy and passed through the microcatheter without occlusion, although some resistance, which seems to have been caused by an increase in viscosity of the TR fluid, was present during the injection. In addition, when injecting TR fluid, there was no reflux into the aorta or migration toward a non-target artery. In all rabbits, the embolization procedure was performed successfully, and post-embolization DSA showed that the renal arterial vessels and renal parenchyma were barely visible (Fig. 6a–d). The baseline characteristics are presented in Table 1.

**Table 1:** Embolization rates and their time course in the four subgroups

Subgroup (Follow-up period, embolic material)		Primary embolization rate (%)			Final embolization rate (%)			Time course of embolization rate	
	*n*	*Mean*	*SD*	*p* value	*Mean*	*SD*	*p* value	%	95% CI [LL, UL]
1-a (7 days, TRF-1)	6	90.4	6.1	0.905	80.7	18.7	0.001**	9.7	[− 9.1, 28.6]
2-a (7 days, TRF-2)	6	91.0	8.2		28.4	19.9		62.6	[40.9, 84.2]^b^
1-b (28 days, TRF-1)	6	90.6	4.0	0.582	94.0	3.5	< 0.001***	− 3.3	[− 10.0, 3.4]^a^
2-b (28 days, TRF-2)	6	88.2	8.5		37.8	15.5		50.4	[30.0, 70.8]^b^

The time-course of embolization rate (%) is the mean difference between the primary embolization rate and the final embolization rate

Primary embolization rate (%) = (1 − the area of renal arterial vessels visualized on post-embolization DSA/ the area of renal arterial vessels visualized on pre-embolization DSA) × 100

Final embolization rate (%) = (1 − the area of renal arterial vessels visualized on follow-up DSA/the area of renal arterial vessels visualized on pre-embolization DSA) × 100

TRF-1 and TRF-2 = type 1 and 2 thermal rheology fluids, respectively. *SD* standard deviation. LL and UL represent the lower limit and upper limit of the confidence interval (CI), respectively

^a^The embolic effect was determined to be persistent, because the 95% CI was < 5%

^b^The embolic effect was determined to be not persistent because the 95% CI was > 15%

**p < 0.01

In the TRF-1 group, the delineation of the renal arterial vessels on follow-up DSA was similar to that on post-embolization DSA (Fig. 6e), while in the TRF-2 group, recanalization was observed on follow-up DSA (Fig. 6f). In terms of the final embolization rate, the embolic effect of TRF-1 was significantly more persistent than that of TRF-2 at both 7 (mean 80.7% [SD 18.7] vs. 28.4% [19.9], *p* = 0.001) and 28 days (94.0% [3.5] vs. 37.8% [15.5], *p* < 0.001) days after embolization (Table 1).

In subgroup 1–b, the embolic effect was persistent (difference between rates − 3.3 [95% CI − 10.0–3.4]; Table 1). However, the embolic effect of TRF-2 was not persistent (difference between rates 62.6 [95% CI 40.9–84.2] and 50.4 [30.0–70.8]; Table 1).

### Pathological analysis

**Fig. 7:**
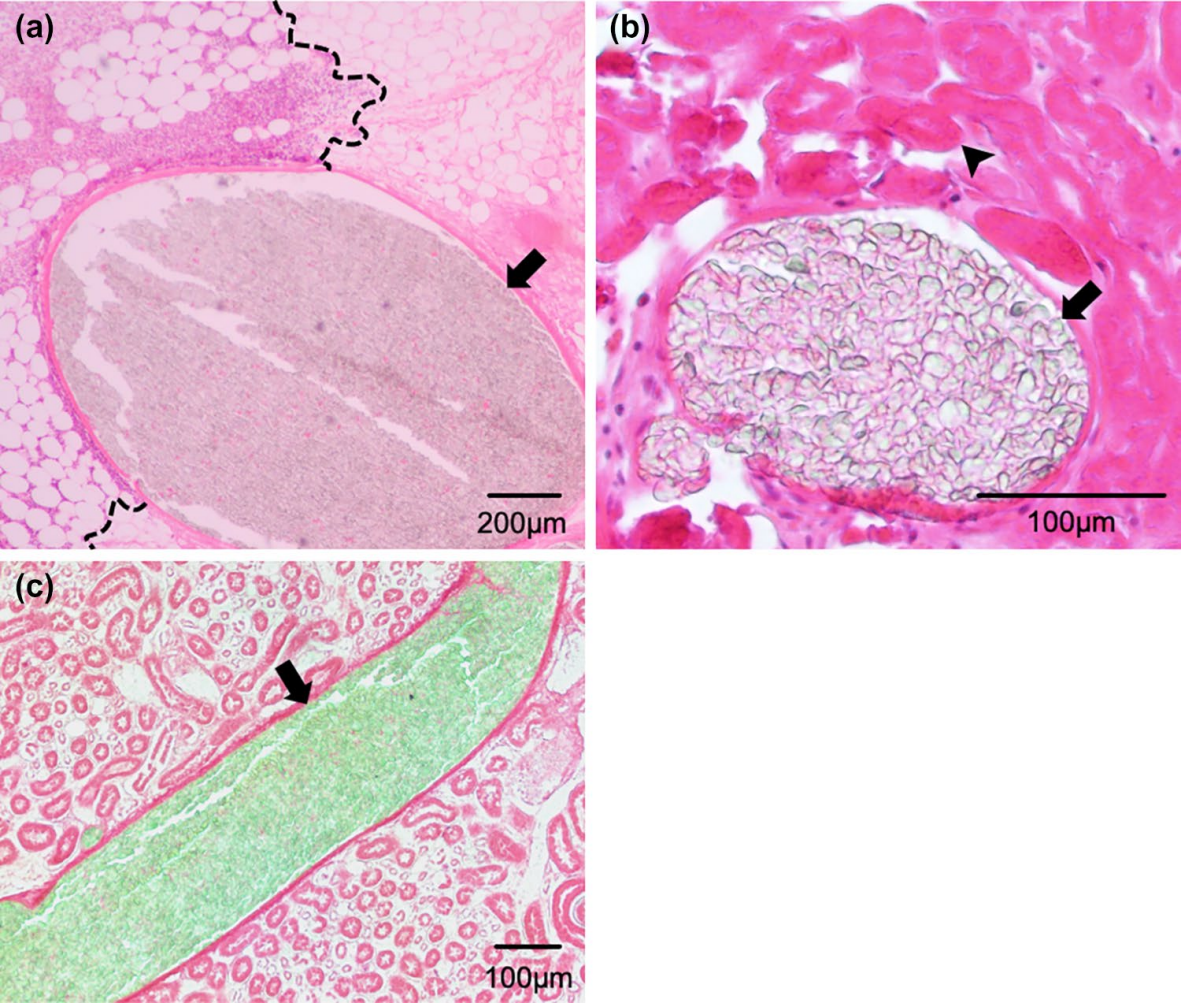
Microscopic findings. The cluster of polyethylene (PE) particles (arrows) containing almost no blood cells or blood clots filled the renal arterial vessels in both type 1 thermal rheology fluid (TRF-1) group (**a**) and type 2 thermal rheology fluid (TRF-2) group (**b**). Inflammatory cell infiltration occurred in the renal sinus area around the vessels (left side of the dotted lines; **a**). Loss of the tubular epithelial nucleus, suggesting tissue necrosis, was observed in the renal parenchyma (arrowhead; **b**). Neither PE particles nor their phagocytosis was seen outside the renal arterial vessels. The largest vessels filled with PE particles were the segmental artery, which was surrounded by renal sinus fat, and the cortical radiate artery inside the renal parenchyma, in TRF-1 group (**a**) and TRF-2 group (**b**), respectively. PE particles (arrow) were continuously distributed from the periphery toward the central side (**c**)

In both groups, the embolized kidneys were atrophied and hard, with irregular edges, and had turned grayish-white in color. Microscopic analysis revealed PE particles occupying the arterial vessels, inflammatory cell infiltration, fibrosis, and necrosis around the vessels (Fig. 7a, b). The area of necrosis was greater in the TRF-1 group than in the TRF-2 group. More specifically, in the TRF-1 group, most tubular epithelial cells were necrotic; in the TRF-2 group, most tubular epithelial cells were necrotic at the cortical margin, rarely necrotic on the hilar side, and the degree of necrosis was intermediate in the middle part. PE particles were present as clusters almost without the intervention of blood cells or blood clots and were distributed from the peripheral small arterial vessels toward the central side in both groups (Fig. 7a–c). Neither PE particles nor evidence of phagocytosis was seen outside the renal arterial vessels. The maximum vessel diameter occluded by PE particles was significantly larger in the TRF-1 group than in the TRF-2 group (mean 870 µm [SD 417] vs. 270 µm [163], *p* < 0.001). In the TRF-1 group, the measured vessels were renal arteries or segmental arteries, surrounded by renal sinus fat (Fig. 7a); in the TRF-2 group, most of the measured vessels were interlobar arteries or more peripheral arteries inside the renal parenchyma (Fig. 7b).

## Discussion

TRF-1 and TRF-2 showed different embolic effect. This result is consistent with previous reports, which demonstrated that the relationship between temperature and viscosity of TR fluid differed depending on the type and ratio of its constituents [2, 5, 6]. In other words, modified TR fluids have new embolic effect. It is suggested that a TR fluid with desired embolic effect customized for each situation can be obtained. TR fluid seems to be safely usable on living organisms under the usual precautions: the pathological changes after embolization with TR fluid were similar to those of renal infarction caused by other etiologies [12–14]; the PE particles themselves remained in the vessels, and no specific adverse reactions were observed.

Although the embolic effect of TRF-2 was not persistent, it may act as a temporary embolic material; however, such usage should be carefully considered. This is because the PE particles in TRF-2 may continue to circulate in the blood vessels for a long period after recanalization of the renal arteries, although the effect of such circulating PE particles on living organisms is currently unknown. Consequently, TRF-2 might gradually embolize micro-vessels in organs throughout the body. It is possible that the viscosity of TRF-2 at rabbit body temperature was not sufficient as an embolic material and that the shearing effect of blood flow gradually washed it away. The PE particles were considered to have low bio-absorption, because no phagocytosis was observed in the pathological images; additionally, non-aggregated PE particles with a size of 7.4 µm are likely to pass through the micro-vessels. In the case of TRF-1, the degree of embolization remained unchanged on follow-up angiography after 28 days. Moreover, the renal arterial vessels, including the proximal arteries such as the renal artery and the segmental artery, were pathologically embolized by aggregated PE. Based on these findings, TRF-1 could be used as a permanent embolic material.

Our findings indicated that TR fluid has a potential as a new embolic material with different characteristics from existing liquid embolic materials. When using absolute ethanol or other radiolucent materials, the real-time state of embolization cannot be observed during procedure. This is associated with the risk of non-target vessel embolization. In contrast, TR fluid has good visibility, can be monitored under fluoroscopy, and was safely injected. Catheter occlusion, catheter adhesion to vessel walls, and the resulting vessel or catheter rupture have been reported as complications of NBCA [15–17], which is classified as the same liquid embolic material as TR fluid. NBCA must be injected quickly because these complications are due to its premature polymerization. Furthermore, NBCA carries a high risk of non-target embolization [17–19]. Training and considerable experience in the effective use of NBCA are required to avoid these complications [15, 20]. In contrast, TR fluid is easy to administer, because it can be delivered without haste and does not block the catheter. In fact, TR fluid did not occlude the microcatheter. Nakai et al. reported that NBCA-lipiodol-ethanol (NLE) has multiple advantages for embolization over NBCA, such as fewer adhesive properties to the catheter and less damage features to the vascular wall [21–24]. TR fluid and NLE appear to have similar advantages for embolization, although they have different embolic mechanisms.

For treating acute arterial bleeding in a coagulopathic state, NBCA, which does not depend on biogenic coagulants, is more useful than other embolic materials, including GS particles and metallic coils [15, 25–28]. Several studies have reported that NBCA was a good embolic material with a high hemostasis rate even in patients with coagulopathy [29–33]. The pathological findings of the current study indicated that the action of TR fluid did not depend on biogenic coagulants, that is, PE particles filled the blood vessels without the aid of thrombi. Therefore, like NBCA, TR fluid should be suitable for patients with coagulopathy. Further examination of TR fluid is warranted to determine its clinical indications, including traumatic bleeding and massive gastrointestinal bleeding.

Our study has several limitations. The first is its small sample size, which may explain why the persistency of the embolic effect could not be determined in subgroup 1-a. Furthermore, inter-individual variation as well as the progression of renal blood vessels atrophy could cause higher embolization rate observed on day 28 than on day 7. However, the use of the minimum necessary animals is recommended for ethical reasons. The second is the short follow-up period. Further investigations should examine longer term embolic effect. The inflexibility of TR fluid is also a limitation. The embolic effect of NBCA can be changed immediately before use by adjusting the mixing ratio with lipiodol, while TR fluid must be already adjusted according to the subject’s body temperature. Finally, our results merely demonstrated that TR fluid embolized the rabbit renal artery: TR fluid was not compared to existing liquid embolic materials such as NBCA and absolute ethanol. Thus, we plan to conduct a comparative study between TR fluid and other embolic materials.

In conclusion, the embolic effect of TRF-1 was more persistent than that of TRF-2, and the persistency depended on the type and ratio of TR fluid constituents.
